# Impact of Tumbling Process on the Toughness and Structure of Raw Beef Meat Pieces

**DOI:** 10.3390/foods10112802

**Published:** 2021-11-14

**Authors:** Konan Charles Aimeric N’Gatta, Alain Kondjoyan, Raphael Favier, Jacques Rouel, Annie Vénien, Thierry Astruc, Dominique Gruffat, Pierre-Sylvain Mirade

**Affiliations:** 1 Université Clermont Auvergne, INRAE, UR370 Qualité des Produits Animaux (QuaPA), 63122 Saint-Genès-Champanelle, France; charles.ngatta@inrae.fr (K.C.A.N.); alain.kondjoyan@inrae.fr (A.K.); raphael.favier@inrae.fr (R.F.); jacques.rouel@inrae.fr (J.R.); annie.venien@inrae.fr (A.V.); thierry.astruc@inrae.fr (T.A.); 2 Université Clermont Auvergne, INRAE, VetAgro Sup, UMR1213 Unité Mixte de Recherches sur les Herbivores (UMRH), 63122 Saint-Genès-Champanelle, France; dominique.gruffat@inrae.fr

**Keywords:** tumbling, muscle fibres, connective tissue, meat toughness

## Abstract

Tenderness is a major factor in consumer perception and acceptability of beef meat. Here we used a laboratory tumbling simulator to investigate the effectiveness of the tumbling process in reducing the toughness of raw beef cuts. Twelve *Semitendinosus* beef muscles from cows were tumbled according to four programs: T1 (2500 consecutive compression cycles (CC), for about 3 h), T2 (6000 CC, about 7.5 h), T3 (9500 CC, about 12 h), and T4 (13,000 CC, about 16 h). The effect of tumbling on the toughness of raw meat was assessed using compression tests (stresses measured at 20% and 80% of deformation ratios) and microscopic observations made at the periphery and centre of meat samples, and compared against non-tumbled controls. Longer tumbling times significantly reduced the stresses measured at 20% and 80% compression rates, which reflected the toughness of muscle fibres and connective tissue, respectively. At the microscopic level, longer tumbling times led to reduced extracellular spaces, increased degradation of muscle structure, and the emergence of amorphous zones. A 12-h tumbling protocol ultimately makes the best compromise between the process time demand and toughness reduction in beef *Semitendinosus* meat pieces.

## 1. Introduction

For the past ten years, the meat industry has tried to provide high-quality meat products to meet rising demand in many countries. Tenderness is a major factor in consumer perceptions and acceptability of meat, as it largely determines eating satisfaction and repurchase decisions. In beef, the huge variability in sensory qualities—particularly tenderness—is the main driver of consumer dissatisfaction [1,2,3].

To improve beef tenderness, the meat industry has developed several processes for tenderizing meat cuts, including enzymatic techniques (injection, infusion, marinating with exogenous enzymes), chemical techniques (injection or marinating with salts or sodium solution), and physical techniques (blade tenderization and tumbling). Blade tenderization and tumbling can improve the tenderness of meat cuts of intermediate tenderness or with high amounts of connective tissue [2]. They cause disintegration of the external surfaces of the meat pieces, disruption of the muscle network, and a release of myofibrillar proteins that increases protein extractability and solubility [4,5,6]. Meat toughness is highly dependent on the state and content of the muscle fibre types and the amount of connective tissue, which determines the ‘background (or baseline) toughness’. The tenderizing effect of the tumbling process is related to the damage to myofibrillar cells caused by mechanical deformations in the meat tissue [7]. Studies have investigated tumbling-induced damage and deterioration at both microscopic and ultrastructural scale and found substantial modifications in muscle tissue [7,8,9,10,11,12,13,14]. 

During tumbling, which is carried out in large rotating drums equipped with baffles or paddles, the meat pieces are subjected to friction with each other or with the baffles, or even with the drum walls, but above all to repeated variations in kinetic energy due to the meat pieces falling and colliding with other pieces, the baffles or the drum walls. All this results in a succession of very short mechanical compressions and, therefore, brief deformations of the meat [15,16]. All these mechanical actions cause changes in the structure of the meat tissue and thus bring about the degradation of the muscle fibres and connective tissue. Tumbling is a well-known meat-industry technique that has the same level of acceptance as blade tenderization on pieces of meat after chilling [3,17]. 

Tumbling conditions and operating procedures vary widely both in industry practice and in the literature. Processing time is usually in the range of 2–16 h, and rotational speed varies from 4 to 12 rpm. Industrial tumbling devices have diameters ranging from 0.5 to 2 m, whereas some pilot-scale tumbling devices have a diameter of less than 0.5 m. The effects of tumbling, especially on the meat deformation, are essentially determined by tumbler size and tumbling duration [18]. Other studies have shown that the tumbling process, especially when carried out over a long period of time, significantly improves meat tenderness by decreasing shear force and hardness values [8,9,19,20], which can enhance the tenderness of less tender cuts of meat [6,10,17,21].

Daudin et al. [16] developed a lab-scale tumbling simulator to reproduce what happens in industrial tumbling devices of various sizes by controlling and characterizing the mechanical deformations undergone by the muscles during tumbling. Here we used this tumbling simulator to: (1) investigate how effectively tumbling reduces the toughness of raw meat pieces cut from beef muscles known to be of intermediate toughness, and (2) to characterize the microscopic damages observed after tumbling in both muscle fibres and connective tissue.

## 2. Materials and Methods

### 2.1. Meat Samples

This study used the beef *semitendinosus* (ST) muscle as it is an intermediate toughness with a high amount of connective tissue [2] and a well-characterized fibre orientation along the length of the muscle. Twenty-four ST muscles were taken from Charolais cow carcasses (52 ± 6 months old): 12 underwent the mechanical treatment described in Section 2.2 and the remaining 12 were used as control samples. These muscles were purchased locally from the same breeder to minimize any effects of variability in husbandry conditions. Before analysis, the muscles were vacuum-packed in polyamide/polyethylene PA 20/PE 70 plastic bags (SAS Boulegon Parry, Clermont-Ferrand, France) with an oxygen permeability <65 g/m^2^/24 h and water transmission <5 g/m^2^/24 h using a Multivac C200 vacuum packing machine (MULTIVAC Sepp Haggenmüller SE & Co. KG, Wolfertschwenden, Germany) and aged for 21 days at 4 °C. The aging time of 21 days was chosen to minimize variability in the mechanical strength of the meat [22]. After aging, the muscles were frozen and stored at −20 °C until analysis. Prior to each experiment, the ST muscles were thawed for 3 days at 4 °C, and then each muscle was trimmed to obtain meat pieces that were 20 cm long and 6.5 cm thick and weighed about 700 g.

### 2.2. Mechanical Treatments

**Figure 1:**
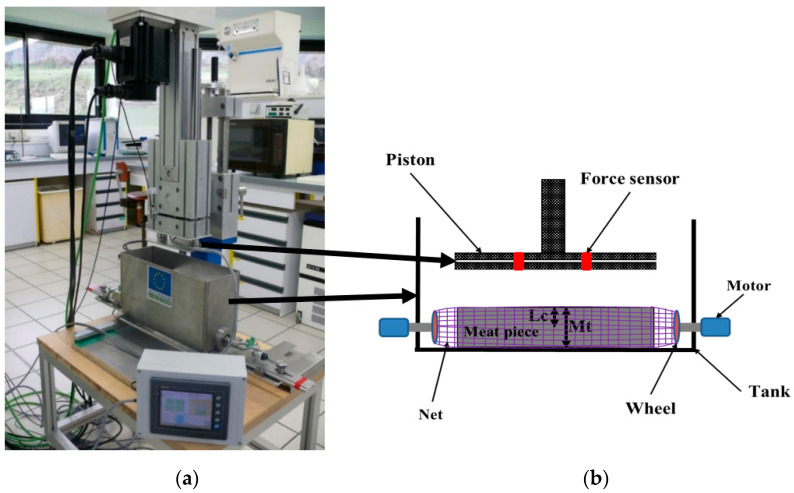
(**a**) Picture of the laboratory tumbling simulator used in this study; (**b**) Schematic view of the system inducing the mechanical deformation of the meat piece in the tumbling simulator.

Before each mechanical treatment, each meat piece was wrapped in an 18-mesh fine elastic net with single weft (SAS Boulegon Parry, Clermont-Ferrand, France) that had negligible mechanical action on the piece of meat. To submit the meat piece to a rotation during the tumbling, the net was attached at both ends to motorized wheels (Figure 1). Mechanical deformations of the meat piece were induced by the linear motion of the piston, which moves up and down at a pre-set frequency. The mechanical treatments of muscles were conducted using the lab-scale tumbling simulator shown in Figure 1 and detailed in [16]. This simulator makes it possible to reproduce what occurs during the meat tumbling process by applying successive mechanical deformations to the muscle that correspond to several hundred cycles of piston compression carried out perpendicular to the length of the muscle. This lab-scale device is designed for tumbling meat pieces at different target compression rates (Crt).

**Figure 2:**
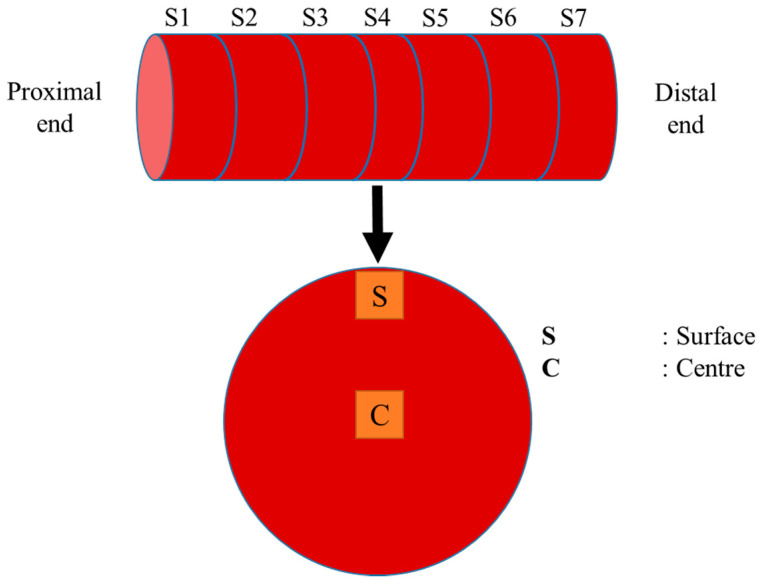
Schematic view of the slice allocation procedure set up for sampling both the tumbled and non-tumbled (NT) ST muscles for microstructure (slice S4) and texture (slices 1–3 and 5–7) analyses.

Considering the thickness of the meat pieces and the limits of the device used, in particular limits relating to the force sensors and the maximum compression stroke of the piston, a Crt of 40% was defined for all the mechanical treatments in combination with a meat compression time of 1.5 s and a time-lapse between two successive deformations of 3 s. The 40% value for Crt was chosen to be high enough to cause structural changes in the meat piece without damaging the lab-scale tumbling device. Tumbler rotation speed was set to around seven rotations per minute (7 rpm). During the trials, the number of successive deformations was fixed at 2500, 6000, 9500 and 13,000, corresponding to treatments T1, T2, T3 and T4, respectively, i.e., experimental times of around 3 h, 7.5 h, 12 h and about 16 h. Each mechanical treatment was performed in triplicate. After each mechanical treatment, the 20-cm-long meat pieces were cut into seven 2 cm- or 3 cm-thick slices (S1, S2, S3, S4, S5, S6, S7) from the proximal end to the distal end, as illustrated in Figure 2. The slice taken at mid-length (2-cm-thick slice S4) was used to prepare samples for microstructural analysis while the other slices were used for textural analysis. The same slice allocation procedure was applied for non-tumbled (NT) control samples.

### 2.3. Textural Measurements

The toughness of raw meat is usually characterized using three different mechanical measurements: compression, Warner-Bratzler (WB) shear force, and texture profile analysis (TPA) [9,23]. Here we used compression tests to evaluate the compressive strength of muscle fibres and connective tissue based on stress measurements at a compression rate of 20% and 80%, respectively [23,24,25]. For these measurements, each 3-cm-thick slice was cut into 10 to 14 samples of rectangular cross-section (1 × 1 × 3 cm^3^). These meat samples were then compressed perpendicularly to muscle-fibre direction using a modified compression cell to avoid transversal elongation of the sample [23]. These compression tests were carried out using a universal testing machine (Instron Model 5543, Instron S.A., Guyancourt, France), and the stress values were then evaluated at 20% and 80% of maximum compression, respectively.

### 2.4. Microstructural and Ultrastructural Studies 

Histological analyses were carried out on the NT and tumbled pieces of raw meat to microscopically identify exactly which meat structures were damaged by the tumbling process. For this purpose, 1-cm^3^ meat samples were extracted from 2-cm-thick slice S4 at two different depths along the diameter of the slice. As detailed in Figure 2, the first level was close to the surface of the piece of meat (Surface, S) and the second (Centre, C) was located 2 cm down from the surface level, corresponding to the centre of the slice. These cubic samples were frozen in isopentane, cooled in liquid nitrogen, and stored at −80 °C until use. To carry out the histological analyses, several 10-µm-thick serial cross sections of fibres were prepared at −20 °C using a cryostat (Microm HM 560, Brignais, France). These histological sections were fixed on glass slides then air-dried at 20 °C. Sections were stained using haematoxylin-eosin-safron (HES) to evaluate the general structure of the muscle tissue, and with picro-sirius red to specifically analyze the endomysium and perimysium structures. To preserve the sections against negative post-stain change, the slides were mounted on a synthetic resin (Eukitt, Kindler GmbH & Co, Freiburg, Germany). Observations were made at 100× magnification on a transmission optical microscope coupled with a digital acquisition kit (Olympus BX61 microscope, Olympus DP 71 digital camera and Cell Sens software, Olympus France SAS, Rungis, France).

Further, to investigate ultrastructural modifications induced by tumbling in each tumbling condition, a 10 × 3 × 3 mm^3^ strip of muscle was immersed in 2.5% glutaraldehyde in 0.1 M sodium cacodylate buffer at pH 5.6 for several weeks. Small pieces (1 to 3 mm^3^) were cut from the strips and post-fixed in 1% osmium tetroxide in cacodylate buffer for 1 h at room temperature. The blocks were dehydrated though an increasing gradient of ethanol concentrations (70%, 95% and 100%) and embedded in epoxy resin (TAAB, Eurobio France). Semi-thin muscle sections of 1 µm thickness cut longitudinally to fibre direction (Reichert-Jung ultramicrotome, Unterschleissheim/Munich, Germany) were placed on a glass slide, stained with Toluidine Blue, and observed by light microscopy with the same Olympus BX 61 microscope as used for cryofixed samples. From the epoxy resin-embedded samples, ultra-thin sections of 90 nm thickness were cut longitudinally to fibre direction (Reichert-Jung ultramicrotome, Unterschleissheim/Munich, Germany), stained with uranyl acetate and lead citrate, and observed under a transmission electron microscope (Hitachi HM 7650, Tokyo, Japan) at 80 kV acceleration voltage. Micrographs were generated using a Hamamatsu AMT digital camera system (Hamamatsu, Japan) coupled to the microscope. Samples were prepared at the INRAE and observed at the Cellular Imaging Center for Health (CICS) laboratory (Université Clermont Auvergne, Clermont-Ferrand, France).

### 2.5. Image Treatments

Quantitative analysis of the 100x-magnified HES-stained cross-sectional images was performed following the procedure described in Astruc et al. [26] with some modifications. Briefly, an average of 8 images per level for each meat piece was used to obtain a better global overview of the muscular structure. Image J software (ImageJ 1.53 g, Rasband et al., National Institutes of Health, Bethesda, MD, USA) was used to separate each image into its 3 monochromatic components: red, green and blue. The green component, providing better contrast between the extracellular spaces area and the muscle fibres, was used to determine the muscle fibre size and extracellular spaces area, expressed in µm^2^. When individualization of the muscle fibres was not feasible, the same procedure was used to quantify the size of the elements detected in the images (amorphous cross-sectional areas). RS-stained images were used to qualitatively evaluate the level of damage to the connective tissue (perimysium and endomysium).

### 2.6. Statistical Analysis

One-way ANOVA was used to determine the effect of treatment intensity (five levels: NT, T1, T2, T3 and T4) on variation in the two stress compression forces evaluated at 20% and 80% of compression rates. Two-way ANOVA was used to determine the effect of mechanical treatments (four levels: NT, T2, T3 and T4) and sampling level (S and C) on changes in extracellular spaces, individual fibres and cross-sectional amorphous-zone areas. Results were expressed as mean ± standard error of the mean. Tukey’s HSD multiple comparison test was then used to determine levels of statistically significance differences (*p*-value < 0.05) among mean values. All statistical analyses were performed using Statistica 13.0 software (Statistica, TIBCO Software Inc., Palo Alto, Santa Clara, CA, USA). 

## 3. Results

### 3.1. Textural Properties 

**Figure 3:**
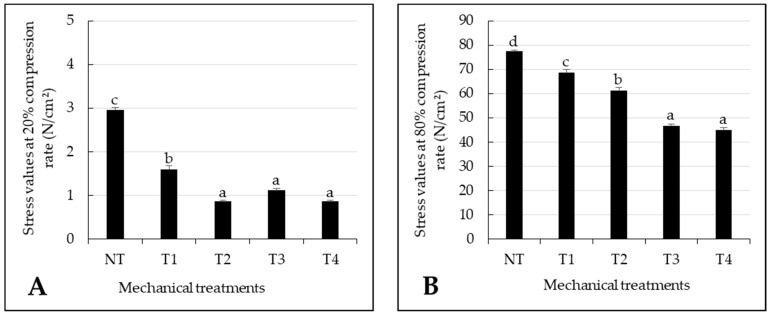
(**A**) Stress values measured at a compression rate of 20% and (**B**) stress values measured at a compression rate of 80% on raw beef *Semitendinosus* meat pieces submitted to five different mechanical treatments: NT (control, no mechanical treatment applied), T1 (2500 compression cycles, about 3 h), T2 (6000 compression cycles, about 7.5 h), T3 (9500 compression cycles, about 12 h) and T4 (13,000 compression cycles, about 16 h). Data are expressed as mean +/− standard error calculated from 300 individual samples prepared from three different meat pieces. Means with different letters (a–d) are significantly different (*p* < 0.05) according to the Tukey test.

Stress values at 20% and 80% compression rates for the four mechanical tumbling programs applied to the ST pieces are presented in Figure 3A,B, respectively. The tumbling programs significantly decreased compression-test stress values at both 20% and 80% compression rates, right from the first level of treatment applied (T1). Thus, compared to the NT sample, the stress values measured at a compression rate of 20% decreased by about 45% with treatment T1 (*p* < 0.05) and by about 70% with treatments T2 to T4 (*p* < 0.05, Figure 3A), suggesting a lower impact of tumbling at short application times. Moreover, we found no significant difference between stress values at the 20% compression rate for the three longer treatments T2, T3 and T4, thus demonstrating a maximal impact of the treatment applied on the stress values at this 20% compression level from treatment T2 upwards.

Mechanical treatment also had a significant effect (*p* < 0.001) on stresses at the 80% compression rate, which decreased with increased tumbling-program time as shown in Figure 3B. The stress values at 80% compression rate were significantly higher (*p* < 0.05) in control samples than all tumbled samples. The 80% compression-rate stress values were also significantly different (*p* < 0.05) between T1, T2 and T3 meat samples (about 11%, 21% and 40%) but not significantly different (*p* > 0.05) between T3 and the longest treatment T4.

### 3.2. Microstructure and Ultrastructure Analyses

**Figure 4:**
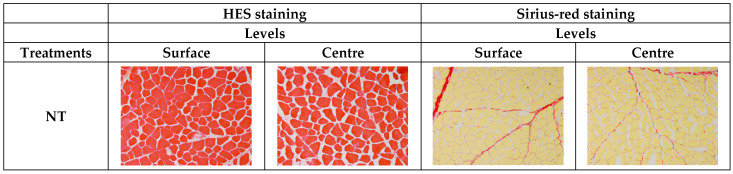

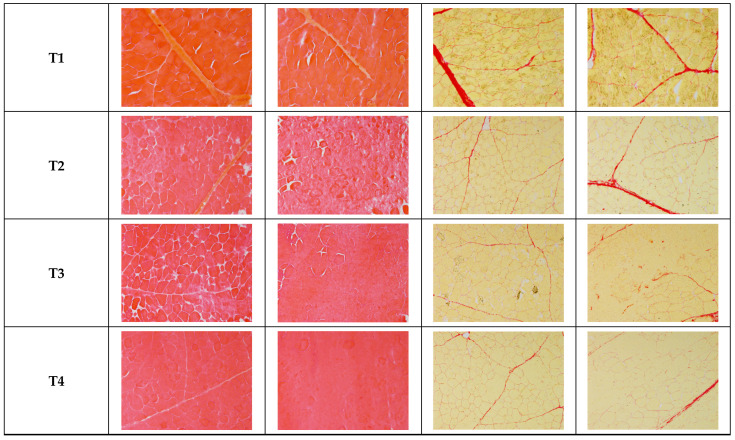
Haematoxylin-eosin-safron and picro-sirius red staining of muscle tissue morphology at two locations, i.e., surface and centre, of raw beef *Semintendinosus* meat pieces having undergone five different mechanical treatments: NT (control, no mechanical treatment), T1 (2500 compression cycles, about 3 h), T2 (6000 compression cycles, about 7.5 h), T3 (9500 compression cycles, about 12 h) and T4 (13,000 compression cycles, about 16 h). Muscle fibres are coloured in red and extracellular spaces are coloured in white for HES staining. Muscle fibres are coloured in yellow and connective tissue is coloured in red for RS staining.

The damage undergone by the meat components during tumbling was characterized at microscopic level on sections stained with haematoxylin-eosin-safron (HES) to observe the general structure of the meat tissue or with picro-sirius red (RS) to observe the damage level of intramuscular connective tissue, perimysium and endomysium (Figure 4). 

HES staining showed that longer tumbling times progressively decreased the extracellular spaces and caused more and more structural damages in tumbled meat pieces (Figure 4). Moreover, compared to NT samples, longer tumbling times caused microstructural changes that differed depending on location in the piece of meat. Structural degradation was more intense and size of the extracellular spaces was even smaller in the centre (C) than at the surface (S) of the tumbled meat pieces. 

Increasing the tumbling time (T1 to T4) promoted a gradual reduction in the extracellular space areas and thus a reduction in the distance between muscles fibres. However, from treatment T2 (6000 compression cycles, about 7.5 h of mechanical treatment), areas started to appear in which it was nearly impossible to distinguish the muscle fibres individually (amorphous zones), probably as a result of the compaction of the muscle fibres.

**Figure 5:**
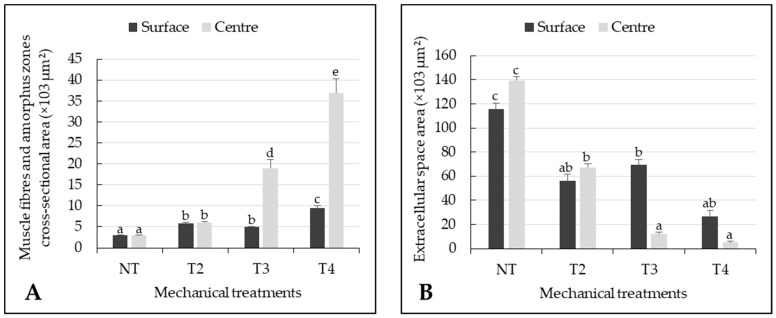
(**A**) Muscle fibre and amorphous-zone cross-sectional area and (**B**) Extracellular space area of raw meat pieces from beef *Semitendinosus* having undergone four different mechanical treatments: NT (control, no mechanical treatment applied), T2 (6000 compression cycles, about 7.5 h), T3 (9500 compression cycles, about 12 h) and T4 (13,000 compression cycles, about 16 h) at two different levels, i.e., surface and centre. Data are expressed as means +/− standard error calculated from 8 images per level from three different meat pieces. Means with different letters (a–e) are significantly different (*p* < 0.05) according to the Tukey test.

Images from experiments NT, T2, T3 and T4 were used to obtain data for characterizing the changes in muscle fibres, amorphous cross-sectional areas (Figure 5A) and extracellular space areas (Figure 5B) according to the sampling depth, i.e., at the meat surface or in the centre. Amorphous-zone areas increased with increasing tumbling time, depending on the depth level investigated: amorphous zone area increased exponentially in the centre of the tumbled meat pieces but only moderately in the surface (Figure 5A). Figure 5B shows that the extracellular space areas were reduced by half in treatment T2 in both surface and centre samples, whereas treatments T3 and T4 led to smaller extracellular spaces in centre samples than surface samples.

A two-way ANOVA showed that mechanical treatment had a significant effect (*p* < 0.001) on the muscle fibres, amorphous-zone and extracellular-spaces cross-sectional areas, whereas location in the meat pieces (surface or centre) only had a significant effect (*p* < 0.001) on amorphous-zone areas. Amorphous-zones areas were significantly higher (*p* < 0.05) in meat pieces from treatments T2, T3, and T4 than control samples. There was no difference in cross-sectional area of amorphous zones between the centre and the surface of the meat pieces from treatment T2, whereas amorphous-zone cross-sectional areas were significantly higher (*p* < 0.05) in the centre than in the surface in treatments T3 and T4 (Figure 5A). Extracellular spaces area was significantly (*p* < 0.05) smaller in tumbled meat pieces from treatments T2, T3 and T4 than control samples. Extracellular spaces areas was not significantly different (*p* > 0.05) between centre and surface in treatments T2 and T4 but significantly higher (*p* < 0.05) in the surface than in the centre in treatment T3 (Figure 5B).

Picro-sirius red staining showed a stronger degradation of the connective tissue network (perimysium and endomysium) in tumbled meat pieces from treatment T2. From treatment T3 onwards, the structural damage became more pronounced and the perimysium became fragmented, distributed in the amorphous areas, and solubilized (Figure 4), with greater effect in the centre than in the surface of the meat pieces.

**Figure 6:**
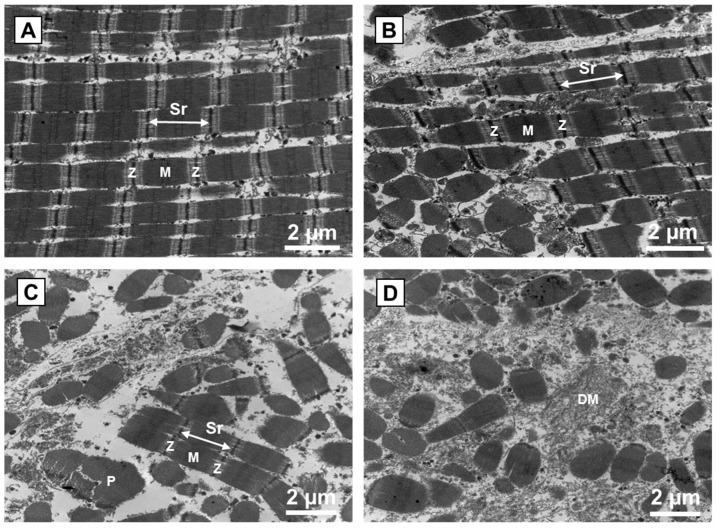
Ultrastructure changes occurred in the centre of raw meat pieces from beef *Semitendinosus* tumbled for different process times. (**A**): NT (control, no mechanical treatment); (**B**): T1 (2500 compression cycles, about 3 h); (**C**): T2 (6000 compression cycles, about 7.5 h); (**D**): T4 (13,000 compression cycles, about 16 h). Sr: Sarcomere; Z: Z-line; M: M-band; P: protein aggregation; DM: depolymerization of the myofilaments.

Transmission electron microscopy made it possible to capture the internal structure of muscle fibres in order to explore the ultrastructural changes in muscle tissue. Figure 6 reports the ultrastructure changes in the centre of the meat pieces according to mechanical tumbling program. Analysis found cracks perpendicular to some muscle fibres (data not shown) due to the 21-day aging time of the meat pieces, which resulted in ultrastructural changes of the myofibrillar structure by cross-fragmentation of the myofibrils at the I-bands and close to the Z-lines. These observations are the result of muscle protein degradations induced by the endogenous meat proteases, which increase their activity with increasing aging time [27]. The tumbling process has an influence on the myofibrillar structure of the meat pieces, especially the sarcomeres, Z-lines and I-bands. 

The application of treatment T1 promoted a disorganization of meat tissue ultrastructure through partial fragmentation of the myofibrils, resulting in isolation of the sarcomeres parallel to the Z-lines and solubilization of some I-bands and Z-lines (Figure 6B). However, from treatment T2 onwards, the changes in the ultrastructure of meat pieces were more severe, with fragmentation of the sarcomeres and increasing solubilization of I-bands and Z-lines, depolymerization of the myofilaments, fragmentation of the sarcolemma, protein aggregation, and transfer of collagen between the damaged myofibrils (Figure 6C,D).

## 4. Discussion

### 4.1. Texture Measurements

The effects of blade tenderization on the textural characteristics of beef has been well documented in the literature [2], but there is little information available on the effect of tumbling on beef meat tenderization, as the tumbling technique is mostly used for cooked ham, marinated pork, and marinated poultry meat [28]. On other side, the mechanical improvement of tenderness through tumbling is secondary to the increase in moisture content and flavor [29]. Some studies have investigated the effect of extending tumbling time on meat tenderness using mechanical measurements [6,9,17,19,30,31,32], but there has been no attempt to study the separate effects of tumbling on muscle fibres and connective tissue as factors in the mechanical properties of beef meat.

Pietrasik and Shand [17] showed on non-injected beef muscles that a pre-intermittent tumbling of 3 h combined with a post-intermittent tumbling of 10 h significantly reduced the WB shear force values of meat samples. They also found that extending the post-injection tumbling period decreased the shear force values of cooked beef muscles. Szerman et al. [8] showed that pre-injection tumbling at 2.5 rpm during 3 h combined with a post-intermittent tumbling at 5 rpm during 10 h significantly reduced the WB shear force values of sous-vide cooked beef compared to non-tumbled beef meat samples. Pietrasik and Shand [6] reported that extended tumbling time had significant effects on hardness and shear forces: cooked roast beef injected at 120% and 140% had lower hardness and WB shear forces values than non-tumbled samples. They also reported that the effect of tenderization was strongly dependent on tumbling time: extending tumbling to 16 h decreased the shear force and hardness of beef samples by 50–60% but was unable to increase cohesiveness. On pork meat, Lachowicz et al. [10] showed that increasing tumbling time up to 12 h led to a decrease in textural (hardness, cohesiveness, chewiness) and rheological (elastic and viscous moduli) properties and thus an increase in textural acceptance of three pork ham muscles. 

Our results on raw beef meat are coherent with the previous literature on cooked beef and pork meat. We demonstrated that, after 3 h of tumbling, there was a reduction in the measured mechanical stress values on the beef ST muscles and therefore a possible improvement in beef meat tenderness. Working on raw beef meat and using compression tests with the two compression rates of 20% and 80% enabled us to separate the time–course changes in mechanical properties of muscle fibres from those of connective tissue [23,24]. Our results show that extending the tumbling time results in an additional decrease in the muscle fibre toughness up to 7 h of treatment while connective tissue strength continued to decrease up to tumbling times of 12 h. These results probably suggest that the decrease in toughness of tumbled beef ST muscles, as evidenced here, could be associated with an improved consumer sensory acceptance of the meat cut from this bovine muscle, as previously mentioned by Lachowicz et al. [10] for pork meat. Interpretations of these variations in mechanical measurements during tumbling warrants discussion on how they relate to the evolution of beef muscle structure, as presented below.

### 4.2. Microstructure and Ultrastructure Characterization

Meat toughness, water holding and water binding are strongly influenced by the microstructure changes in meat tissue [9]. The intensity of muscle structure damage is hugely dependent on the method and process used to alter the structure of the meat products [11]. Several authors have already studied the effects of the tumbling process on meat microstructure and ultrastructure [7,8,10,11,12,13]. Theno et al. [13] showed that increasing massaging time to 24 h completely disrupted the muscle fibres. They found that short tumbling times (1 to 2 h) resulted in the degradation of some muscle fibres at microscopic level, whereas after 4 h of continuous massaging, the muscle fibres were fully disrupted. Cassidy et al. [7] showed that the tumbling process damaged the cell membranes both on the surface and in depth, thus ultimately resulting in a more tender meat. Our results here converge with this pattern, as we observed, a reduction in the size of the extracellular spaces after 3 h of tumbling, and it became nearly impossible to individualize the muscle cells after 7.5 h because of the formation of amorphous zones. The reduction in extracellular spaces probably resulted from the compression effect due to action of the piston on the meat tissue during mechanical treatment, which compacted the muscle fibres and disrupted connective tissue. 

Lachowicz et al. [10] showed that the TPA hardness of tumbled pork cuts was strongly correlated with thickness of the perimysium and endomysium (coefficients of 0.88 and 0.90, respectively), and that decreasing both the perimysium and endomysium thickness led to a reduction in the hardness of these meat cuts. The results obtained here in beef meat are in agreement with Lachowicz et al. [10] on pork.

The development of amorphous zones, which is representative of a greater degradation of the muscle fibres, has already been observed by Astruc et al. [26] in the case of beef ST muscles incubated for 5 days in 1 M NaCl brine. These authors attributed their observations to the ‘salting-in’ phenomenon during which increasing NaCl concentration promotes protein solubilization. However, here the meat pieces were not brined or salted, but only tumbled. Nevertheless, several published studies [8,12,17] have shown that tumbling provoked the deterioration and disruption of the muscle tissue and then the extraction and solubilization of myofibrillar proteins on the surface and inside the meat pieces. These phenomena get stronger as tumbling time continues [33].

Using a meat activator, Tyszkiewicz and Jakubiec-Puka [11] reported that the mechanical tenderization of porcine *biceps femoris* muscles induced several changes in meat tissue by destroying many of the linkages between the muscle fibres and myofibrils. These changes may be responsible for the reduction in toughness of pork meat due to the damage of muscle fibres and connective tissue. The authors asserted that the increase in the number of kinked and twisted fibres induced by mechanical treatment would be due to degradation of the endomysium, indicating a deterioration of both myofibrillar and connective tissue components. In addition, Theno et al. [12] studied ultrastructural changes in ham *rectus femoris* muscles during massaging and observed that continuous massaging for 4 h caused a longitudinal separation of the myofibrils; after 24 h of continuous massaging, an excessive fibre disruption occurred, thus leading to a longitudinal shredding of the fibres and, ultimately, to a detachment of the myofibrils. These results are consistent with the ultrastructural changes observed in our study. However, the damage levels observed here were more severe than those reported in these previous studies, especially when tumbling time was prolonged, i.e., number of deformation cycles increased. This is probably due to the different kind of equipment and method used to mechanically tenderize the meat pieces and thus disrupt the muscle structure. Indeed, the lab-scale tumbling simulator used in this study reproduced mechanical treatments similar to those at work in industrial tumblers. This lab-scale device delivers mechanical deformation of meat pieces through brief mechanical compression of muscle tissue [16]. The development of amorphous zones and the reduction in extracellular spaces in our study is almost certainly the consequence of the transmission of compression and frictional energies to muscle tissue components (muscle fibres and connective tissue), which translated into severe degradation of the myofibrils, fragmentation of the sarcomeres at the I-bands and Z-lines, depolymerization of the myofilaments, fragmentation of the sarcolemma, and the transfer of collagen between the damaged myofibrils. Indeed, although the tumbling times (number of compression cycles) differed, the compression rate target and rotation speed were the same for all mechanical treatments. The levels of muscle structure damage were therefore dependent on total energy dissipated in the meat pieces, which increased with increasing tumbling times and led to intense disruption of the muscle tissue. Moreover, the meat pieces used in this experiment had been aged for 21 days, and the proteolysis status of the myofibrils was therefore high. The high degree of mechanical damage observed here was possibly facilitated by the prior deterioration of the structural muscle proteins by endogenous enzymes and the increase in their activity in the meat tissue for longer tumbling times [27].

Analysis of the meat structure during tumbling (Figure 4 and Figure 6) showed that the reductions in meat toughness observed at tumbling times shorter than 7.5 h can be mainly attributed to degradation of muscle fibres and, to a lesser extent, disruption of the connective tissue. The evolutions observed for the longest tumbling times are more likely explained by a greater degradation of the connective tissue, thus contributing to a decrease in ‘background’ or baseline toughness.

## 5. Conclusions

Using a previously designed lab-scale tumbling simulator with a compression ratio of 40% enabled raw beef meat to be mechanically tenderized. Extending tumbling times to 16 h led to weakening of the protein network, characterized by a reduction in the compressive strength of muscle fibres and connective tissue by 45–70% and 11–40% on average, respectively. Quantitative analysis of the histological images highlighted a reduction in extracellular spaces and the appearance of amorphous zones where it became practically impossible to distinguish individual muscle cells at the longest tumbling times. Microstructure and ultrastructure imaging demonstrated that the decrease in meat toughness at long tumbling times was related to increased degradation of both muscle fibres and connective tissue.

Based on this evidence, the tumbling process can be used for tenderizing pieces of raw beef exhibiting intermediate initial tenderness, and in the longer term for developing new beef meat products, as tumbling can be coupled with marinating. Applying 9500 consecutive compression cycles (about 12 h) with a compression ratio of 40% appears to offer the best compromise between reduction in meat toughness and duration of the tumbling program. Further studies are needed to investigate whether the improvement in the tenderness found here on raw beef meat carries through to after cooking, and whether the improvement detected here by the mechanical compression tests is also perceptible to consumers.

## Data Availability

Not applicable.
